# Adaptation of a Community Clinical Linkages Intervention to the COVID-19 Pandemic: A Community Case Study

**DOI:** 10.3389/fpubh.2022.877593

**Published:** 2022-06-22

**Authors:** Kiera Coulter, Maia Ingram, Abby Lohr, Carlos Figueroa, Gloria Coronado, Cynthia Espinoza, Maria Esparza, Stacey Monge, Maria Velasco, Lee Itule-Klasen, Magdalena Bowen, Ada Wilkinson-Lee, Scott Carvajal

**Affiliations:** ^1^Mel and Enid Zuckerman College of Public Health, University of Arizona, Tucson, AZ, United States; ^2^Department of Community Internal Medicine, Mayo Clinic, Rochester, MN, United States; ^3^Yuma County Public Health Services District, Yuma, AZ, United States; ^4^Pima County Health Department, Tucson, AZ, United States; ^5^El Rio Community Health Center, Tucson, AZ, United States; ^6^Sunset Community Health Center, Yuma, AZ, United States; ^7^Department of Mexican American Studies, University of Arizona, Tucson, AZ, United States

**Keywords:** community-clinical linkages, community health worker, community-based participatory research, adaptation, COVID-19

## Abstract

In this community case study, we describe the process within an academic-community partnership of adapting UNIDOS, a community health worker (CHW)-led community-clinical linkages (CCL) intervention targeting Latinx adults in Arizona, to the evolving landscape of the COVID-19 pandemic. Consistent with community-based participatory research principles, academic and community-based partners made decisions regarding changes to the intervention study protocol, specifically the intervention objectives, participant recruitment methods, CHW trainings, data collection measures and management, and mode of intervention delivery. Insights from this case study demonstrate the importance of community-based participatory research in successfully modifying the intervention to the conditions of the pandemic and also the cultural background of Latinx participants. This case study also illustrates how a CHW-led CCL intervention can address social determinants of health, in which the pandemic further exposed longstanding inequities along racial and ethnic lines in the United States.

## Introduction

The coronavirus disease 2019 (COVID-19) is an unprecedented threat to global public health that has drastically changed public health research agendas, and the ways in which we conduct health intervention research. With the restrictions imposed to mitigate COVID-19 transmission, health intervention research planned prior to the pandemic has become challenged and constrained. Finding ways to modify health intervention delivery has been critical because health promotion, specifically for chronic disease prevention, is paramount due to the known link between chronic disease and increased risk of COVID-19 morbidity and mortality (1). Chronic disease inequities along racial and ethnic lines have produced similar disparities in COVID-19 infections and death (2). By necessity, social determinants of health (SDOH) should be a component of health intervention research, particularly to address health equity in ethnic-minority communities and those most affected by COVID-19 (3). Research conducted within and in partnership with historically marginalized communities offers a chance to respond to these underlying inequities in areas such as housing, employment, and access to healthcare during the pandemic. The objective of this case study is to describe how community-based participatory research (CBPR) methods facilitated the adaptation of a chronic disease intervention (which we refer to herein as the UNIDOS intervention) to the COVID-19 pandemic within Mexican-origin communities in the U.S.-Mexico border region of Arizona, and positioned our academic-community partnership to respond to the emerging SDOH needs that became more pronounced as a result of the ongoing pandemic.

CBPR is driven by consistent collaboration between researchers, community members, and community entities to determine the research objectives, collect data, analyze the results, disseminate the results, and create plans for sustainability (4). Thus, CBPR involves working from the ground up and engaging alongside communities to identify their health priorities, challenges, and potential solutions (5). In our case, the Arizona Prevention Research Center (AzPRC) launched the UNIDOS intervention in the beginning of 2020, and originally intended to focus on chronic disease inequities among Latinx communities by addressing their SDOH needs, enhancing participants' social support, and leveraging social networks. The process of adapting UNIDOS to the COVID-19 landscape was driven by partnerships between the AzPRC, federally qualified health centers (FQHCs), and local county health departments (LCHDs) in response to the disproportionate impact of COVID-19 transmission and the socioeconomic fallout of the pandemic in Latinx communities.

The pandemic further clarified the importance of addressing SDOH issues within health promotion interventions. Given the timing of implementing UNIDOS in relation to the pandemic, the intervention needed to be responsive to evolving SDOH needs and COVID-19 safety precautions. In this community case study, we describe the process through which our academic-community partnership used CBPR to adapt the protocol of the UNIDOS intervention (which at the time of writing this article the intervention is ongoing) during COVID-19, focusing on Mexican-origin communities along the U.S.-Mexico border. The description of the adaptation is based upon internal conversations with UNIDOS personnel who represent academic institutions, FQHCs, and LCHDs.

## Context: Setting and Population

The UNIDOS intervention prioritizes adults of Latinx origin residing within four Arizona counties: Yuma County, Pima County, Maricopa County, and Santa Cruz County. These counties are proximal to the U.S.-Mexico border, and are also home to a significant proportion of the Mexican-origin population within Arizona. Prior to the pandemic, residents of the border region were already facing challenges to their SDOH needs which negatively affected their health status. Border residents are twice as likely to live in poverty, face barriers to healthcare access, and experience higher rates of unemployment than the population of any U.S. state (6). Additionally, the increased militarization of the U.S.-Mexico border region (i.e., increased U.S. Customs and Border Patrol personnel and expansion of the border wall) contributes to a hostile anti-immigrant environment which subjects migrants and Latinx residents to maltreatment and immigration-related profiling often stemming from racist nativism (7). The onset of the COVID-19 pandemic worsened existing disparities across Arizona and the U.S.-Mexico border. At certain points during the course of the pandemic, Arizona led the globe in COVID-19 cases per capita (8). The contextual factors within the border region specifically made residents more vulnerable to COVID-19 infection, death, as well as the socioeconomic consequences of the pandemic. Farm workers, both in Southern Arizona and the broader U.S., experienced high rates of COVID-19 infection (9). Restrictions on non-essential travel at the U.S.-Mexico border strained businesses in the border region (10). Undocumented migrants, in Southern Arizona and across the U.S., were excluded from COVID-19 relief (i.e., stimulus checks) on the basis of their citizenship status (11).

The pandemic also impacted the operations of the UNIDOS intervention's community partners. The UNIDOS intervention is facilitated by community health workers (CHWs) situated within FQHCs and LCHDs in each of the counties. At the beginning of the pandemic, patient volumes within some of the partnering FQHCs dropped and clinic personnel, including CHWs, were laid off or furloughed. Additionally, some of the health promotion activities (e.g., diabetes management classes, exercise classes, etc.) were paused due to social distancing constraints and personnel changes. Within the LCHDs, CHWs were immediately involved in various COVID-19 response efforts (e.g., COVID-19 contact tracing). The UNIDOS intervention was adapted within this landscape of instability experienced by residents and health systems of Southern Arizona.

## CBPR Partnerships and the UNIDOS Intervention

The academic and community partners of the AzPRC have a more than 25 year history of initiatives aimed to enhance the impact of CHWs in community and clinical settings. The activities of the AzPRC are based upon shared leadership with the Community Action Board (CAB), consisting of organizational and community members of Southern Arizona's border region. The overall objectives of the AzPRC in partnership with the CAB are to advance health in border communities, reduce ethnic disparities in health, promote the CHW model for health promotion, study chronic disease interventions with partners in the border region, and interrelate these activities with community development and advocacy of systems or policy change. The collaborative research endeavors of the AzPRC are based upon the tenets of CBPR, which are embedded into our guiding principles. To this end, the AzPRC uses various tools to continuously evaluate its partnership activities and the engagement of community agencies, to ensure equity within the research collaborations (12). As part of UNIDOS, the CAB's research committee meets monthly over the course of the project to guide research activities while also defining and collaborating in research dissemination (i.e., peer-reviewed publications, conference presentations, practice-based tools). The CAB's research committee consists of the academic and community personnel (i.e., CHWs and CHW supervisors within partnering FQHCs and LCHDs) who are directly involved in implementing the UNIDOS intervention. The process of adapting UNIDOS took place within these monthly meetings over Zoom, as well as by giving the four UNIDOS sites autonomy to make local modifications based on the conditions of the pandemic in their communities. As the severity of COVID-19 worsened in 2020, and localities across the U.S. implemented restrictions to combat COVID-19 transmission, the research committee initiated discussions regarding if and how to proceed with conducting the UNIDOS intervention.

UNIDOS is a CHW-led, SDOH-focused intervention based upon a community-clinical linkages (CCL) model within each partnering county. CCL models are a health systems approach to addressing SDOH that extend the continuum of care from clinical settings to the community and are designed to improve patient access to community and public health services (13). The intention behind embedding CHWs within the CCLs was to highlight their effectiveness in addressing SDOH needs within community and clinical settings. CHWs, as frontline public health workers, possess competencies and skills that are necessary to creating and sustaining successful CCL programs, such as strong relationships with community residents and entities, as well as being trusted members of the communities they serve (13). Within the original design of UNIDOS, CHWs at FQHCs identify eligible participants based upon chronic disease risk, SDOH needs, or other health promotion needs/interests. CHWs housed within LCHDs then deliver the intervention which focuses on leveraging community resources to respond to participants' SDOH needs and building social networks. The CHWs based out of LCHDs provide referrals and facilitate access to health promotion resources (e.g., chronic disease self-management programs, physical activity programs, tobacco cessation programs) and SDOH resources (e.g., housing support, citizenship classes, academic and career resources) within participants' local communities. CHWs also provide ongoing social support and foster participants' social networks as guided by the sociocultural resilience model [SRM; 14]. SRM posits that cultural factors among Latinx communities lead to health-promoting social processes that increase healthy behaviors, bolster health resiliencies (i.e., tangible support, communal coping, and resource sharing), and ultimately contribute to health advantages that characterize Latinx populations relative to other racial and ethnic groups in the U.S. (14). Given CHWs' social relationships and involvement in social networks among communities, the SRM provides a potential role for CHWs to further direct their activities in ways that are health protective and culturally responsive, while improving coordination within CCLs. Prior to COVID-19, CHWs were going to facilitate individual and group-based social support sessions to help participants develop coping and problem-solving strategies, assist participants in identifying and accessing their social network, and foster peer support in group settings.

## Adaptation of UNIDOS

The following section describes how the academic-community partnership, specifically the CAB's research committee, modified the research process and components of the UNIDOS intervention to meet the challenges of the COVID-19 pandemic. As previously mentioned, CBPR necessitated that changes were made in collaboration with the community partners based upon their observations and experiences within the four UNIDOS sites. In response to the pandemic, the CAB's research committee made changes to the intervention objectives, participant recruitment methods, CHW training, intervention delivery, and data collection/management within the research protocol, as summarized in Table 1.

**Table 1:** Summary of the adaptation of the UNIDOS intervention.

**UNIDOS intervention component**	**Original UNIDOS intervention**	**Adapted UNIDOS intervention**	**Adaptation rationale**
Intervention objectives	• Reduce chronic disease risk • Enhance social support • Strengthen social networks	• Address participants' SDOH challenges that were caused or exacerbated by COVID-19 • Enhance social support	• The CAB's research committee recognized that addressing participants' SDOH could serve to respond to participants' pandemic-related challenges while also reducing chronic disease risk • Enhancing social support remained a primary objective of the intervention given the social isolation participants were experiencing due to COVID-19 • During the pandemic, participants could not interact within their social networks as they had before, thus the UNIDOS CHWs increasingly focused on providing social support
Participant recruitment	• CHWs recruit participants in-person within community and clinical settings (e.g., patient waiting rooms, health fairs, churches, existing health promotion programs within their host organizations)	• CHWs innovated new recruitment strategies including leveraging partnerships with local agencies, websites/social media announcements, newspaper announcements, local radio and news broadcasts, and COVID-19 efforts (e.g., contact tracing phone calls, vaccination sites)	• The CAB's research committee and university's human subjects research leadership determined that in-person participant recruitment should not take place at venues where social distancing or COVID-19 protective measures were not in place
UNIDOS CHW training	• AzCHOW conducts UNIDOS CHW training in-person, and focuses on core competency training (i.e., skills intrinsic to CHWs' scope of practice) and creates a peer-sharing network to allow CHWs to share best-practices in the field	• AzCHOW conducted UNIDOS CHW trainings virtually • AzCHOW continued to incorporate core competency training, mentorship, and a peer-sharing network, but also included pandemic-specific topics (e.g., promoting COVID-19 vaccinations) and discussions of how the pandemic impacts CHWs (e.g., CHW mental health, demands on CHWs during COVID-19) • CHWs received pandemic-related trainings from their host organizations (e.g., contact tracing)	• CHWs were impacted not only as individuals during the pandemic but also by the increased severity of needs among the participants they served • Some CHWs were integrated into pandemic response efforts within their community, and their host organizations provided relevant trainings to perform these activities
Intervention delivery	• CHWs conduct the baseline and follow-up assessments with participants in-person and/or over the phone • CHWs refer participants to in-person health promotion classes in LCHDs • CHWs connect participants to SDOH services • CHWs facilitate individual and group-based social support sessions	• CHWs conducted baseline and follow-up sessions over the phone or online (e.g., Zoom) • CHWs referred participants to virtual health promotion classes (if available within the LCHD) • CHWs connected participants to an expanded range of SDOH services due to creation of COVID-19 relief resources • CHWs provided social support on an individual basis over the phone or online	• The CAB's research committee decided that the intervention needed to be carried out remotely to ensure CHWs' and participants' safety within the pandemic • Health promotion classes within LCHDs were offered virtually or canceled • CHWs linked participants to a wider range of SDOH resources as local, state, and national COVID-19 relief services were created in response to the pandemic • Social support groups were not possible due to social distancing guidelines
Data collection and management	• CHWs facilitate the UNIDOS assessment at baseline, 3-months, and 6-months documenting participants' demographics, physical and emotional health, social support and social networks, sociocultural resilience, and SDOH needs as well as engagement in referred health promotion/SDOH services • CHWs complete a monthly follow-up form to document participants' health status, their challenges/progress, and action plan	• A COVID-19 questionnaire was added to the UNIDOS assessment • The PRAPARE (SDOH instrument) was expanded to capture a broader range of SDOH • Local, state, and federal services and resources that addressed the needs documented within the COVID-19 and PRAPARE questionnaires were integrated into the REDCap database	• The CAB's research committee added and expanded measures within the UNIDOS assessment to document participants' wider range of concerns and needs that emerged during the pandemic • The community partners suggested integrating information regarding SDOH and COVID-19 relief services into the REDCap database so that CHWs could link participants immediately to needed resources while also capturing data on CHWs' referrals

*CHW, community health worker; CAB, community action board; SDOH, social determinants of health; LCHD, local county health department; AzCHOW, Arizona Community Health Workers Association*.

### Intervention Objectives

The intensification of COVID-19 transmission and the disproportionate burden of COVID-19 on Latinx communities prompted initial discussions regarding the relevance of UNIDOS during the pandemic. The original objectives of the UNIDOS intervention were to (1) reduce chronic disease risk and (2) enhance social support and strengthen social networks to improve participants' health status. For the first objective, UNIDOS reduces chronic disease risk via CHWs addressing participants' SDOH needs through connections to local services and resources. There was agreement among the community partners that addressing SDOH was fundamental to the intervention. Moreover, the partners recognized that participants were in immediate need of COVID-19 information and education (e.g., how to follow COVID-19 safety precautions, where to get tested, etc.) while also confronting pressing socio-economic challenges (e.g., housing) and were unaware of resources that could assist them. Collaborative discussions between the academic and community partners generated consensus that tackling participants' SDOH challenges via the UNIDOS CCL model could simultaneously address participants' pandemic-related challenges and chronic disease risk. Thus, the primary objective of UNIDOS remained the same, but the mechanism through which chronic disease risk would be reduced (i.e., SDOH) would also serve to address participants' pandemic-related needs that either emerged during or were exacerbated by COVID-19. For the second objective, the CAB's research committee felt strongly that enhancing participants' social support (via their interactions with the CHWs) was still a crucial component of the intervention due to increasing isolation experienced by community members as well as the ongoing uncertainties and stressors of the pandemic. Many participants were unable to see family members and friends for prolonged periods of time, and were experiencing anxiety and fear surrounding COVID-19 and economic challenges. As such, increased social support from the CHWs would not only be a mechanism for improved health status but also address an immediate impact of the pandemic and bolster participants' emotional wellbeing. Furthermore, CHW social support was critical given that participants could not tap into their social networks as before and thus could not be a leverageable component of the intervention. In sum, the partners felt that the activities of the UNIDOS intervention (i.e., connecting participants to health promotion resources, linking participants to SDOH resources, and maximizing social support) could address participants' pandemic-related needs, and the original objectives of UNIDOS could be applied in the pandemic context.

### Participant Recruitment

Prior to COVID-19, the CHWs were going to recruit participants solely through in-person interactions in community and clinical settings (i.e., existing health promotion programs, churches, health fairs, patient waiting rooms, etc.). However, the CAB's research committee agreed that direct face-to-face contact with participants could no longer continue under the threat of COVID-19. To facilitate participant recruitment safely, each site innovated community-based recruitment strategies. For example, CHWs increasingly relied upon alliances with local agencies who served the target population (e.g., Mexican consulate, faith organizations) to help reach eligible participants, and these agencies also shared information regarding the UNIDOS intervention on their social media and website pages. CHWs also utilized local and bilingual newspaper, radio, and news broadcasts as mediums to disseminate study information within their communities. Another strategy was to pair participant recruitment with COVID-19 efforts. For example, one site distributed UNIDOS flyers with consent forms to individuals being monitored in their cars after receiving COVID-19 vaccinations at vaccine points of distribution (PODs). Additionally, another site followed-up with people who had received a COVID-19 contact tracing call to inform community members regarding UNIDOS and conduct the consent process over the phone if individuals were eligible and interested in participating.

### CHW Training

The Arizona Community Health Outreach Worker Association (AzCHOW) has been responsible for training CHWs facilitating the UNIDOS intervention. Originally the training was intended to primarily focus on core competencies (i.e., skills intrinsic to CHWs' scope of practice) and create a peer-support network to allow the CHWs to share best-practices in the field. More experienced CHWs have utilized the training time to mentor newer CHWs, for example through role-playing exercises. The training also includes information on human subjects' protocols, data collection, and data management. Due to COVID-19, AzCHOW has incorporated pandemic-specific trainings on topics such as the impact of COVID-19 on farmworkers and COVID-19 vaccine promotion. The increasing demands placed on CHWs due to participants' complex pandemic-related needs also prompted AzCHOW to include discussions of CHWs' mental health. For example, the AzCHOW facilitators led training sessions on stress management and self-care, avoiding burn out, meditation, and grounding yourself. This CHW training has served as a space for CHWs to discuss the stressors they are experiencing during the pandemic as residents of their communities and facilitators of UNIDOS.

The collaborating FQHCs and LCHDs also provided pandemic-related training for the CHWs. As previously mentioned, the CHWs in some counties were mobilized into pandemic-response efforts at the outset of COVID-19, and they underwent contact tracing and isolation guidelines trainings. Also, some of the CHWs received suicide prevention training in response to increased depression and mental health struggles experienced by their community's residents.

### Intervention Delivery

UNIDOS is based upon a CCL framework where CHWs connect participants to (1) health promotion classes offered by LCHDs (e.g., chronic disease self-management programs), (2) SDOH services within the community (e.g., academic and career resources, citizenship classes), and (3) CHW-facilitated social support. Due to COVID-19, modifications were made to the intervention delivery as well as the resources that participants were being referred to. For service referral, the available health promotion classes that CHWs were linking participants to transitioned to a virtual format in some sites (e.g., exercises classes over Zoom). Also, the SDOH services expanded as needs emerging from the pandemic proliferated and/or were exacerbated, and local, state, and federal relief resources were created in response. Relief resources included services such as emergency housing, food banks, workforce development, and free internet access. The CHWs also provided participants with information regarding COVID-19 testing sites, safety precautions (e.g., how COVID-19 is transmitted, social distancing and masking guidelines), and how to access COVID-19 vaccines. In some instances, the CHWs helped participants directly make COVID-19 vaccination appointments once they were eligible. Linking participants to health promotion classes, SDOH services, and/or COVID-19 resources required the CHWs to spend time coaching individuals on how to utilize virtual technologies such as email, Zoom, or telehealth platforms. Lastly, the social support activities of UNIDOS had to be restricted to individual interactions between the CHW and participant (over the phone or virtually), as in-person one-on-one and peer support groups did not allow for maintaining adequate social distancing. Within the monthly follow-up visits, CHWs continued to discuss participants' abilities to access resources but have given increased focus to providing emotional support to help participants cope with the additive stressors related to COVID-19 (e.g., disease transmission, economic challenges, and social isolation). In some cases, CHWs also provided support around grieving when a participant experienced the loss of a friend or family member from COVID-19.

### Data Collection and Management

To assess the efficacy of and fidelity to the UNIDOS intervention, CHWs facilitated a questionnaire encompassing a variety of qualitative and quantitative indicators. CHWs administered the questionnaire with participants at baseline, 3, and 6-months, and also completed a follow-up form at each monthly visit (baseline−6 months) to document participants' health status, their challenges and/or progress made regarding their wellness goals, and the action plan to address their needs. CHWs input data into the REDCap database. The questionnaire, prior to any changes, assessed participants' demographics, physical and emotional health, social support and social networks, sociocultural resilience and SDOH needs. CHWs also noted participants' engagement in referred SDOH services at each follow-up visit. Given that the focus of the UNIDOS intervention had shifted to helping participants navigate the COVID-19 landscape, study assessments were both added and modified. The academic researchers recommended questions that were being used in other COVID-19 research projects to the CAB's research committee, and the research committee members provided input and revised the new study assessments to ensure that the questions reflected the challenges experienced by community residents.

First, a COVID-19 questionnaire was added to the questionnaire to document participants' concerns and needs regarding COVID-19, encompassing health concerns (e.g., having the correct information about COVID-19 and how to protect yourself), social concerns (e.g., social isolation), economic concerns (e.g., loss of employment), and internet access. Secondly, per the suggestion of the community partners, we integrated information regarding relevant SDOH services and resources into the COVID-19 questionnaire and Protocol for Responding to and Assessing Patients' Assets, Risks, and Experiences (PRAPARE) instruments (a standardized assessment tool measuring SDOH within community health settings). In this way, we transformed the UNIDOS database into not only a tool to collect data but also an active resource hub. In REDCap, our team applied branching logic to the COVID-19 and PRAPARE questionnaires so that resources (including agency names, phone numbers, description of services, and relevant notes) appeared on the screen based on participants' responses. Because the resources were often county-specific as community-level organizations and institutions (e.g., churches, food banks, schools) sought to address local needs, we created two separate databases for each set of counties (i.e., a database for Pima and Yuma counties and another for Maricopa and Santa Cruz counties), with each database containing resources specific to those counties. Because resource information was rapidly changing, the CHWs, UNIDOS program coordinator, and the AzPRC database manager communicated regularly to update the database. The flow of the resource instruments (COVID-19 and PRAPARE forms) within the REDCap databases was structured as follows: If a participant responded in a manner indicating needing a resource (e.g., responding “Yes” to “Are you worried about losing your housing?”), county-specific and/or state- wide and/or national resources would then populate underneath the question. In this example, the CHW would see a list of rent assistance or shelter resources appear within the database. The CHW would then indicate whether a specific resource was provided to the participant by clicking a “Yes” or “No” radio button to facilitate more robust action planning detailed in subsequent notes and record keeping. This responsive format had two advantages: (1) it gave CHWs the ability to immediately provide and document needed referrals and (2) it saved CHWs' time because they did not need to search for resources or sift through unapplicable information (e.g., resources pertaining to other counties or obsolete resources).

## Results

At the time of writing this article, the UNIDOS intervention remains ongoing. To date, CHWs in Pima and Yuma counties have successfully navigated a cohort of participants through the full 6-months of the adapted intervention. Maricopa and Santa Cruz counties have recently initiated implementing UNIDOS (December 2021). Additionally, while the intervention continues to be implemented, the process of adapting UNIDOS provides important lessons learned for practitioners and researchers.

### SDOH Service Referral

As of December 2021, 125 participants had enrolled in the UNIDOS intervention across Pima and Yuma counties. By this date, CHWs in Yuma and Pima counties had made service referrals across a broad spectrum of needs based upon participants' responses to the PRAPARE and COVID-19 forms. For example, in response to housing needs, the CHWs made 48 referrals to housing shelters, and 59 referrals to eviction/rent/utility assistance services. CHWs also made 15 referrals to unemployment services (e.g., Department of Economic Security). To address food insecurity, CHWs made 26 referrals to local food pantries. Overall, CHWs made 197 service referrals across Yuma and Pima counties for mental health (e.g., counseling, suicide prevention hotlines), representing the most prevalent service referral.

### Lessons Learned

Successfully adapting the UNIDOS intervention required overcoming several challenges, which stemmed from uncertainties regarding the relevance of UNIDOS and difficulties in its implementation during the pandemic. These challenges, the facilitators/assets utilized to overcome them, and key lessons learned are summarized in Table 2. Overall, the use of CBPR was critical to effectively modifying UNIDOS. Because each county partner was represented within the CAB's research committee, they were positioned to modify the intervention collaboratively with the academic researchers based upon their knowledge of what was important and feasible given the evolving realities of COVID-19 on the ground.

**Table 2:** Summary of the challenges, facilitators, and lessons learned.

**Challenges**	**Description of challenge**	**Facilitators**	**Lessons learned**
Relevance of UNIDOS during COVID-19	• The academic-community partnership questioned the relevance of UNIDOS given the intervention's original focus on chronic disease, the demands placed on community partners to respond to COVID-19, and the growing severity participants' SDOH challenges	• CBPR research approaches • CCL framework	• Via the CAB research committee, community partners were positioned to modify the intervention based upon their knowledge of what was important and feasible • In the context of COVID-19, strict fidelity to the original study protocol was not practical • The CCL model positioned CHWs to effectively link community residents to a network of local service providers to address pandemic-related challenges
Implementing UNIDOS during COVID-19	• After switching UNIDOS to a virtual format, CHWs confronted having to recruit and engage participants in socially-distanced ways	• CHWs' community networks and partnerships • CHWs' interpersonal competencies	• CHWs leveraged partnerships with local media and community entities to recruit participants • CHWs were able to maintain participant rapport over the phone by paying close attention to the tone of the participants' voices, listening for verbal cues, and increasingly focusing on how they verbally respond
	• Engaging in UNIDOS and accessing services during COVID-19 required internet familiarity, and many UNIDOS participants did not know how to use and/or have internet access	• CHWs' coaching abilities	• CHWs are able to effectively teach participants how to use the internet/internet platforms
	• CHWs needed to respond to a broader range of SDOH needs due to COVID-19, and capturing data on CHWs' referrals was challenging because of the dynamically changing landscape of COVID-19 relief resources	• Collaboration between CHWs' and AzPRC personnel • CHWs' and CHW supervisors' community networks and partnerships • REDCap database	• Keeping the database up to date so that CHWs could refer participants to active services required constant communication between the CHWs, the AzPRC program coordinator, and the database manager • Partnerships with local agencies, developed by the CHWs and their supervisors, were crucial to service referral within the intervention • The REDCap database served dual purposes as an active resource hub for the CHWs to make real-time service referrals, and to capture data on service referral

*CCL, community-clinical linkage; CBPR, community-based participatory research; CHW, community health worker; CAB, community action board; SDOH, social determinants of health; AzPRC, Arizona Prevention Research Center*.

Once the CAB's research committee determined that UNIDOS needed to be conducted online and/or over the phone, the CHWs were able to develop alternative strategies to continue participant recruitment while maximizing participant benefit of the adapted virtual intervention. Additionally, the academic researchers needed to be flexible with the UNIDOS protocol, given the demands placed on community partners as they were responding to and/or being impacted by COVID-19 in real time. In other words, due to the pandemic, strict fidelity to the original UNIDOS protocol was not practical. Lastly, the CCL model positioned CHWs to effectively link community residents to a network of service providers to address pandemic-related challenges. To that end, partnerships with local agencies, developed by the CHWs and their supervisors, have been crucial to participant recruitment and service referral within the intervention. For example, one CHW supervisor had a relationship with an emergency housing organization which a CHW leveraged to provide housing to a participant facing eviction.

Also, while CHWs have been able to effectively establish rapport with participants, the CHWs initially felt challenged by the socially-distanced interactions with participants which were primarily over the phone. If a participant was in emotional distress, the CHWs were not able to see participants' facial expressions (if speaking via phone), nor hug or touch them. Some CHWs were concerned that this degree of separation might result in participants perceiving the interaction with the CHW as cold, especially given the importance of interpersonal warmth in Latinx cultures. To better gauge the emotional state of the participant, some CHWs adapted by paying close attention to the tone of the participants' voices and listening for verbal cues. One CHW has also increasingly focused on the way in which she verbally responds to participants over the phone to ensure that participants are feeling supported despite the lack of in-person interaction. In spite of these communication barriers, UNIDOS CHWs have been able to provide participants with ongoing social support during the pandemic despite not being able to meet face-to-face with them. The UNIDOS CHWs and their supervisors note that participants greatly appreciate the phone calls from the CHWs and having someone check-in on them, especially as the pandemic was intensifying and family visitation was restricted. There were instances when the follow-up phone calls were participants' only source of social interaction that particular day, increasing the importance and impact of CHW follow-up.

Another issue CHWs encountered was participants' unfamiliarity with internet and computer technology, especially among older participants. Many participants did not know how to use and/or have access to a computer, tablet, or smart phone. If they did have computer access, CHWs noted that elderly participants were intimidated by using Zoom, and sometimes were hesitant to learn how to use it. Also, during the pandemic, accessing services increasingly moved to online platforms, and CHWs took on the additional task of providing intensive coaching on how to use the internet or create an email account so participants could successfully utilize a resource (e.g., making a COVID-19 vaccination appointment or scheduling a doctor's appointment). However, in other instances, the CHWs have acknowledged that virtual programming has been useful in reaching participants who normally confront transportation or childcare barriers. At the time of writing this article, the UNIDOS community partners are considering offering face-to-face CHW-participant interactions when safety permits, while continuing to offer remote participation options (e.g., phone and Zoom).

Lastly, a unique feature of the UNIDOS intervention is the dual use of the REDCap database as a data collection platform and active resource hub. CHWs used the data collected from the COVID-19 and PRAPARE assessments specifically to document participants' pandemic and broader SDOH-related needs, as well as to guide service referral. Capturing data on CHWs' referrals to specific services was challenging at times because of the dynamically changing landscape of COVID-19 relief resources. The database manager continuously modified the REDCap database based upon the creation and termination of relief services, most of which were specific to a UNIDOS county site. Available resources were consistently in flux over the course of the pandemic as new services were created or not sustained. Keeping the database up to date so that CHWs could refer participants to active services required constant communication between the CHWs, the AzPRC program coordinator, and the database manager. Thus, in essence, the REDCap database served as an active resource hub for the CHWs as they assisted participants in navigating the COVID-19 landscape by linking them to service providers. Also, given the success and adoption of the REDCap databases for UNIDOS, it is acting as a template for future CBPR projects.

## Discussion

The emergence of COVID-19 necessitated adapting the protocol and delivery of the UNIDOS intervention. This case study exemplifies how an academic-community partnership utilized CBPR to successfully modify UNIDOS to meet the challenges created or worsened by the COVID-19 landscape. Because the design and procedures of UNIDOS were dynamically changing alongside the conditions of the pandemic, the academic team and community partners continuously reevaluated the study objectives, participant recruitment methods, CHW training modules, intervention procedures, and study assessments to adequately respond to COVID-19. Moreover, during the pandemic CHWs successfully linked participants to a variety of SDOH resources, suggesting their effectiveness within pandemic settings.

### Implications for Practice

Our case study underscores the centrality of CBPR for successfully adapting health intervention studies during the COVID-19 pandemic. Per CBPR, communities co-design the intervention and the process of intervention design can be flexible and iterative. These two methodological aspects of CBPR were critical in modifying UNIDOS during the pandemic. CBPR elevated the community partners as leaders within the research team, which was important for adapting UNIDOS as the community partners possessed expertise on participants' needs and the realities of COVID-19 on the ground. Additionally, CBPR provided the space from which the academic-community partnership could jointly assess the relevance and practicality of the UNIDOS intervention as the pandemic evolved, and make ongoing changes to the intervention protocol. Thus, to maximize the responsiveness of community-based interventions to the pandemic and beyond as communities recover, participatory research methods will remain a critical tool for research teams engaged in these types of health intervention studies.

The case study also emphasizes the importance of having CHWs implement health promotion strategies as part of pandemic response. Within UNIDOS, the CHWs' connections with local agencies were critical to addressing participants' SDOH needs which were caused or worsened by COVID-19. CHWs' position as trusted members within their communities was essential to reaching and engaging Latinx adults in the CCL model. By cultivating strong rapport with participants, the CHWs were able to provide needed education on COVID-19 transmission, mitigation strategies, testing, and vaccines (among other activities). This is noteworthy given that ethnic-minority populations have been disproportionately affected by the pandemic, both in terms of COVID-19 infection/death as well as the pandemic's socioeconomic effects. As a result, there has been a need for public health professionals who can successfully reach vulnerable communities to disseminate COVID-19 information and connect them to resources addressing their SDOH needs; our case study suggests that the CHW workforce is ideally suited to fulfill these gaps in the pandemic response. Echoing this observation, the Centers for Disease Control and Prevention (CDC) has recognized that CHWs are well positioned to assist communities most impacted by COVID-19 (e.g., ethnic-minority communities) and their involvement could be expanded to help these communities move toward health equity (15).

## Conclusion

The UNIDOS intervention, based upon a CHW-led CCL model, was originally designed to address chronic disease risk and emotional wellbeing among Latinx communities in the border region of Arizona. The COVID-19 pandemic necessitated the adaptation of UNIDOS, and this case study demonstrates the importance of CBPR in modifying a health intervention to address SDOH needs created or worsened by the global pandemic. Because of the trusting relationships within the longstanding academic-community partnership, the academic team and community partners were able to make needed changes to the intervention objectives, methods of participant recruitment, CHW training, intervention delivery, and data collection/data management practices in order to successfully respond to COVID-19. This case study also exemplifies how a CHW-led CCL intervention was leveraged as a means of pandemic-response to tackle participants' SDOH needs, which were caused or exacerbated by COVID-19. Because COVID-19 further exposed longstanding inequities along racial and ethnic lines in the U.S., elevating interventions like UNIDOS is necessary to more intentionally connect ethnic-minority communities to needed SDOH services and resources.

## Data Availability Statement

The raw data supporting the conclusions of this article will be made available by the authors, without undue reservation.

## Ethics Statement

The studies involving human participants were reviewed and approved by University of Arizona. The patients/participants provided their written informed consent to participate in this study.

## Author Contributions

KC led the development of the manuscript and conducted the interviews with the academic and community partners involved in implementing the UNIDOS intervention. KC and MI collaborated on the conceptualization of the community case study. KC, MI, and AW-L developed the interview guide utilized to interview the academic and community partners. AL, CF, GC, CE, ME, SM, MV, LI-K, and MB participated in the interviews and provided their insights regarding the process of adapting UNIDOS and specific modifications that were made given their role in the research project. All authors contributed to the article and approved the submitted version.

## Funding

This publication is a product of a Health Promotion and Disease Prevention Research Center supported by Cooperative Agreement Number (DP19-001) from the Centers for Disease Control and Prevention.

## Author Disclaimer

The findings and conclusions in this report manuscript are those of the author(s) and do not necessarily represent the official position of the Centers for Disease Control and Prevention.

## Conflict of Interest

The authors declare that the research was conducted in the absence of any commercial or financial relationships that could be construed as a potential conflict of interest.

## Publisher's Note

All claims expressed in this article are solely those of the authors and do not necessarily represent those of their affiliated organizations, or those of the publisher, the editors and the reviewers. Any product that may be evaluated in this article, or claim that may be made by its manufacturer, is not guaranteed or endorsed by the publisher.
